# White matter microstructure contributes to age-related declines in task-induced deactivation of the default mode network

**DOI:** 10.3389/fnagi.2015.00194

**Published:** 2015-10-09

**Authors:** Christopher A. Brown, Jonathan G. Hakun, Zude Zhu, Nathan F. Johnson, Brian T. Gold

**Affiliations:** ^1^Department of Anatomy and Neurobiology, University of KentuckyCollege of Medicine, Lexington, KY, USA; ^2^Department of Rehabilitation Sciences, University of KentuckyLexington, KY, USA; ^3^Sanders-Brown Center on Aging, University of KentuckyLexington, KY, USA; ^4^Magnetic Resonance and Imaging and Spectroscopy Center, University of KentuckyLexington, KY, USA

**Keywords:** diffusion tensor imaging (DTI), fMRI, deactivation, aging, default mode network (DMN)

## Abstract

Task-induced deactivations within the brain’s default mode network (DMN) are thought to reflect suppression of endogenous thought processes to support exogenous goal-directed task processes. Older adults are known to show reductions in deactivation of the DMN compared to younger adults. However, little is understood about the mechanisms contributing to functional dysregulation of the DMN in aging. Here, we explored the relationships between functional modulation of the DMN and age, task performance and white matter (WM) microstructure. Participants were 117 adults ranging from 25 to 83 years old who completed an fMRI task switching paradigm, including easy (single) and difficult (mixed) conditions, and underwent diffusion tensor imaging (DTI). The fMRI results revealed an age by condition interaction (β = −0.13, *t* = −3.16, *p* = 0.002) such that increasing age affected deactivation magnitude during the mixed condition (β = −0.29, *t* = −3.24 *p* = 0.002) but not the single condition (*p* = 0.58). Additionally, there was a WM by condition interaction (β = 0.10, *t* = 2.33, *p* = 0.02) such that decreasing WM microstructure affected deactivation magnitude during the mixed condition (β = 0.30, *t* = 3.42 *p* = 0.001) but not the single condition (*p* = 0.17). Critically, mediation analyses indicated that age-related reductions in WM microstructure accounted for the relationship between age and DMN deactivation in the more difficult mixed condition. These findings suggest that age-related declines in anatomical connectivity between DMN regions contribute to functional dysregulation within the DMN in older adults.

## Introduction

Functional neuroimaging studies have demonstrated that some brain regions show lower activation during the performance of attention-demanding cognitive tasks compared to simpler baseline conditions (Shulman et al., 1997; Gusnard and Raichle, 2001; Raichle et al., 2001). Such task-induced deactivations tend to occur within the brain’s default mode network (DMN), which includes medial prefrontal cortex, posterior cingulate cortex/precuneus, lateral portions of temporal and parieto-occipital cortices and hippocampus (HC) (Gusnard and Raichle, 2001; Raichle et al., 2001; McKiernan et al., 2003; Persson et al., 2007). The DMN contributes to a wide variety of internally-generated thought processes (Gusnard and Raichle, 2001; Buckner et al., 2008). As such, the capacity to deactivate the DMN is thought to have important functional significance during externally-directed cognitive tasks (McKiernan et al., 2003; Persson et al., 2007).

Consistent with this possibility, younger adults tend to show greater deactivation in the DMN as the task becomes more cognitively demanding (McKiernan et al., 2003; Persson et al., 2007; Park et al., 2010; Sambataro et al., 2010). Interestingly, several studies have reported that older adults show similar magnitude of DMN deactivation as younger adults during simple cognitive tasks, but less deactivation during more difficult active tasks (Persson et al., 2007; Park et al., 2010; Sambataro et al., 2010). These findings suggest that older adults may be less able to regulate functional response in DMN cortical regions as cognitive resources are increasingly required by the active task (Persson et al., 2007; Sambataro et al., 2010). Reduced deactivation of the DMN during task performance could thus be a significant contributor to older adult difficulties on attention-demanding tasks.

Despite the broad potential relevance of this issue, little remains known about how deactivation of the DMN may track with increasing age and its neurobiological sequelae such as reductions in task performance and brain structure. This is in part because previous work using parametric manipulations has focused solely on comparisons of younger and older adult groups. However, by treating age continuously, it is possible to examine multivariate relationships with other continuous variables, such as neuroimaging measures of potential structural mechanisms contributing to deactivation of the DMN.

We hypothesized that white matter (WM) microstructure may be a potential contributor to deactivation patterns in the DMN for several reasons. First, because WM provides a means for electrochemical signaling across large-scale brain networks, age-related reductions in WM microstructure would be expected to influence the coordinated functioning of brain regions within organized networks such as the DMN (Andrews-Hanna et al., 2007; Vidal-Piñeiro et al., 2014). Second, recent studies have confirmed several underlying WM connections between various portions of the DMN (Greicius et al., 2009; van den Heuvel et al., 2009; Teipel et al., 2010; van Oort et al., 2014). Finally, there is evidence that the WM microstructure measure of fractional anisotropy (FA) is correlated with BOLD magnitude in task-positive regions during cognitive task performance (Persson et al., 2006; Madden et al., 2007; Daselaar et al., 2013; Hakun et al., 2015; Zhu et al., 2015) and with functional connectivity within portions of the DMN (Andrews-Hanna et al., 2007; Khalsa et al., 2013).

Here we tested the hypothesis that age-related reductions in WM connectivity could limit the capacity to divert resources away from the DMN when those resources are increasingly required by the active task. An fMRI task switching paradigm was used that included two simple perceptual judgment tasks (color and shape judgments) made on stimuli that were identical in each task condition. Difficulty was manipulated by asking participants to either repeat the same task throughout a block or switch (pseudorandomly) between judgment types, placing increased demands on cognitive control processes (Kramer et al., 1999; Kray and Lindenberger, 2000). We also investigated the effects of age, DMN WM microstructure, and task-induced deactivation on task performance across the two conditions. Finally, mediation analysis was used to determine whether WM microstructure accounts for the age-related decline in DMN deactivation capacity as cognitive demands increase.

## Materials and Methods

### Participants

There were a total of 117 participants in the present study. Participants were community-dwelling adults between the ages of 25 and 83 (mean = 49.7 ± 17.4) with normal or corrected-to-normal vision. Written informed consent was obtained from each participant under an approved University of Kentucky Institutional Review Board protocol. Exclusion criteria were color blindness, a major head injury, stroke, a neurological or psychiatric disorder, high blood pressure, diabetes, use of psychotropic drugs, or presence of metal fragments and/or metallic implants contraindicated for MRI.

Participants completed measures of fluid intelligence and digit span, which are known to correlate with task-switching performance (Kray and Lindenberger, 2000). Fluid IQ was measured using the Cattell Culture Fair Intelligence Test (Cattell and Cattell, 1960) because this test assesses non-verbal intelligence associated with perceiving inductive relationships in shapes and figures, processes relevant to the perceptual task-switching paradigm employed in the present study. Digits forward (DF) and backward (DB) were measured using The Digit Span Subtests of the Wechsler Memory Scale (WMS III) (Wechsler, 1997). Socioeconomic status was assessed using the Hollingshead 2-Factor Index of Social Position (ISP), which is based on educational and occupational achievement (low scores = higher SES) (Hollingshead, 1957). Demographic information is provided in Table 1.

**Table 1:** **Demographic and performance scores**.

	Mean ± SD
N	117
Age	49.7 ± 17.4
M:F	52:65
Education	16.7 ± 2.8
ISP	25.3 ± 13.7
IQ	124.5 ± 18.9_115_
DF	10.6 ± 2.1_113_
DB	10.2 ± 2.6_113_
Single Acc	97.7 ± 2.6
Mixed Acc	95.3 ± 4.6
Single RT	725.3 ± 147.9
Mixed RT	945.3 ± 203.9

ISP, Hollingshead Index of Social Position; IQ, Cattell Culture Fair Intelligence Test; DF, digit span forward; DB, digit span backward; Single and Mixed refer to conditions in the fMRI experiment; Acc: accuracy RT: reaction time. Values listed are mean ± SD. Values for IQ and DB are age-scaled scores. If score values were missing, the number of participants used in the computation is shown as a subscript.

### Image Acquisition

Imaging data were collected on a 3T Siemens TIM scanner at the Magnetic Resonance Imaging and Spectroscopy Center at the University of Kentucky. High resolution anatomic images were acquired using a magnetization-prepared rapid echo gradient-echo (MPRAGE) sequence (repetition *time*_(TR)_ = 1690 ms, echo *time*_(TE)_ = 2.65 ms, flip angle = 12°, 1 mm isotropic voxels). T2*-weighted functional volumes were collected using a gradient-echo echo-planar imaging (EPI) sequence (33 interleaved slices, TR = 3000 ms, TE = 30 ms, flip angle = 83°, field of *view*_(FOV)_ = 224 mm^2^, matrix = 64 × 64, 3.5 mm isotropic voxels). Diffusion tensor images (DTI) were collected using a double spin-echo EPI sequence (TR = 6900 ms, TE = 105 ms, flip angle = 90°, FOV = 224 mm^2^, in-plane resolution = 1.75 × 1.75 mm voxels, 40 contiguous 3 mm-thick axial slices) with 36 non-collinear encoding directions (*b* = 1000 s/mm^2^) plus 5 images without diffusion weighting (*b* = 0 s/mm^2^, b0).

### fMRI Task Paradigm

Participants completed a task-switching paradigm described in detail elsewhere (Zhu et al., 2015). Briefly, participants performed one of two possible perceptual judgments on every trial. On color task trials, participants decided if a stimulus was red or blue. On shape task trials, participants decided if a stimulus was a circle or square. Participants were asked to respond as quickly and as accurately as possible. During single task blocks the same judgment was made on every trial for the duration of the block (e.g., all color trials), while in mixed task blocks shape and color judgments alternated pseudorandomly with a 50% probability of repeating/switching between judgments on consecutive trials. There were three runs, each consisting of four task blocks and five fixation blocks. Task blocks consisted of 20 trials (totaling 60 s per block) for each of the single or mixed task conditions. During fixation blocks (lasting 30 s), participants were instructed to look at a centrally presented cross-hair (+). One run consisted of two single blocks of each color and shape judgments, and the other two runs consisted of one single block for each judgment and two mixed blocks.

### fMRI Preprocessing and Analyses

fMRI Expert Analysis Tool (FEAT) v6.0.0, part of the FMRIB Software Library (FSL; Smith et al., 2004), was used for fMRI data preprocessing and statistical analysis. Data were first motion corrected, smoothed with a 9 mm Gaussian kernel and high-pass filtered at 100 s. fMRI data was then co-registered to each individuals high resolution structural scan using boundary-based registration (Greve and Fischl, 2009). The high resolution structural image was co-registered to MNI 2 × 2 × 2 mm space using an initial linear registration followed by nonlinear warping (using FNIRT; Andersson et al., 2007). These transformation parameters were then applied to the functional data, which was re-sliced to 2 mm isotropic voxels during non-linear warping into MNI space.

Single and mixed blocks were modeled separately and convolved with a double-gamma hemodynamic response function. First-level analysis involved contrasts for color and shape (single) blocks compared to fixation and mixed blocks compared to fixation for each participant for each run. Parametric maps from each first-level analysis (Runs 1, 2, and 3) were then carried into a second-level fixed effects model where the results of each first-level model were combined into a single beta-map per condition. Higher-level (group) analyses were performed using FMRIB’s Local Analysis of Mixed Effects (FLAME).

### Defining a Common DMN Network

A common deactivation network was defined across participants to identify DMN structures of functional relevance to individuals across the age range of our sample. Peak regions of deactivation were identified through a voxelwise contrast of fixation > single task across all participants. Significant clusters of peak deactivation in this contrast were masked at a voxel-wise FWE corrected *p* < 0.05 threshold. The single (i.e., easier) condition was chosen for the contrast given previous evidence of stronger age-differences during more difficult task conditions (Persson et al., 2007; Sambataro et al., 2010). (However, it should be noted that the baseline fixation > mixed task condition contrast produced essentially the same results in our data; peaks within one voxel of those in the fixation > single condition). The overall DMN mask was created by placing 5 mm radius spheres around the peak voxel in each cluster surviving the above criteria in the fixation > single contrast. Percent signal change within the overall DMN mask was then extracted for each participant using Featquery.

### Diffusion Tensor Imaging Preprocessing

FMRIB’s Diffusion Toolbox (FDT) v3.0 was used for all DTI preprocessing and analyses. Preprocessing steps performed on raw images involved eddy current correction, brain extraction, and motion correction using a 12-parameter affine transformation to the b0 images. DTIFIT was then used to compute a tensor model and eigenvalues (*λ*_1_, *λ*_2_, *λ*_3_) within each voxel. These eigenvalues were used to calculate FA images, which were used as input for tract-based spatial statistics (TBSS).

### Tract-Based Spatial Statistics

Each participant’s FA image was co-registered to the FMRIB58_FA 1 mm standard space template using tract-based spatial statistics (TBSS; Smith et al., 2006), as described in detail in our previous work (Johnson et al., 2012). Briefly, non-linear voxel-wise registration was used to transform images into MNI space, where FA images were averaged to generate a mean FA image. The mean FA image was subsequently used to create a common WM tract skeleton. This skeleton was thresholded at FA > 0.2 in order to minimize partial volume effects after warping across all participants. Each participants FA image was subsequently projected onto the FA skeleton in order to account for residual misalignments between participants after initial registration.

### Probabilistic Tractography

Probabilistic tractography was performed to identify the WM pathways connecting the eight individual fMRI clusters forming the overall “network-level” DMN-fMRI mask (described in Defining a Common DMN Network). Prior to tractography, the fMRI-based cortical seeds were registered to each participant’s native diffusion space. The first step in this process was to transform the cortical seeds from MNI 2 × 2 × 2 mm space to MNI 1 mm^3^ space using FLIRT. Then the inverse of the non-linear warp from each participant’s FA image to MNI 1 mm^3^ space (described in Tract-Based Spatial Statistics) was generated and applied to register all fMRI-based cortical seeds from MNI 1 mm^3^ space to each participant’s diffusion space.

Tractography was performed using FSL’s Bayesian Estimation of Diffusion Parameters Obtained using Sampling Techniques (BEDPOSTX) and probabilistic tracking (PROBTRACKX2) tools (Behrens et al., 2003, 2007). BEDPOSTX (Behrens et al., 2003) was run using a 2-fiber model to determine a probabilistic diffusion model in each voxel, which was used as the input for probabilistic tractography. PROBTRACKX2 (Behrens et al., 2007) was run in network mode using modified Euler streamlining. A total of 5000 samples were generated from each voxel in each seed with a curvature threshold of 0.2 (approximately ± 80°), a step length of 0.5 mm with a maximum of 2000 steps, and a fiber volume threshold of 0.01. The Harvard-Oxford Subcortical Atlas brainstem mask was used as an exclusion mask to avoid descending pathways. Successful streamlines were those originating in a voxel within one fMRI-based cortical seed and passing through a voxel in another fMRI-based cortical seed without entering the exclusion mask.

A streamline image was generated showing all successful tracts, which was then registered to MNI 1 mm^3^ standard space using the non-linear transform described above. Each individual’s streamline image was divided by the total number of samples generated (# of samples = # voxels in seed regions * 5000) to create a proportion image. The value of each voxel in the proportion image indicates the proportion of successful streamlines that passed through that voxel. Before creating a group mean image, each participant’s proportion image was divided by the total number of successful streamlines generated, or waytotal, as a way to account for differences in “trackability” between each participant’s diffusion data. A group mean image was then calculated from the average of all participants’ waytotal-normalized proportion maps. The group proportion image was thresholded at 0.1% of total streamlines passing through a given voxel in order to generate a group mask of successful streamlines. The group mask was restricted to those voxels within the mean FA skeleton to limit the effects of partial voluming. Mean FA values were then extracted from the skeletonized “DMN-WM” mask for each participant.

### Statistical Analyses of Behavioral, fMRI and DTI Data

Statistical analyses were carried out in SPSS 22 (IBM Statistics, Chicago, IL). Three main analyses were carried out. The first two analyses were intended to assess the contributions of relevant predictors on: (1) DMN deactivation magnitude; and (2) task performance. These analyses used mixed-effects General Linear Models (GLM), which examined fixed-effects while accounting for subject-specific variance. The first set of GLMs investigated the between-subject effect of either age or FA, the within-subject effect of condition (single or mixed), and the age/FA × condition interaction on DMN deactivation magnitude. The second set of GLMs investigated the between-subject effect of either age, FA, or DMN deactivation magnitude, the within-subject effect of condition, and the age/FA/deactivation × condition interaction on task performance (accuracy or reaction time (RT) separately). Standardized beta-values are reported for each effect and *t*-statistics were used as the inferential statistic to determine significance of each effect in the presence of the others. Those effects with *p* < 0.05 were considered significant. Significant interactions were investigated further by performing linear regression within each condition separately.

The final set of analyses used mediation models to test the hypothesis that DMN WM microstructure accounts for age-related changes in DMN deactivation magnitude. Mediation analysis based on multiple linear regression was performed using macros (PROCESS; Hayes, 2013) that simultaneously estimate all paths between variables and indirect effects. PROCESS utilizes bootstrapping and generates Monte Carlo confidence intervals to allow for statistical inferences. Indirect effects having 95% confidence intervals not crossing zero were accepted as significant mediation effects.

## Results

### Functional Deactivations Across Participants

**Figure 1:**
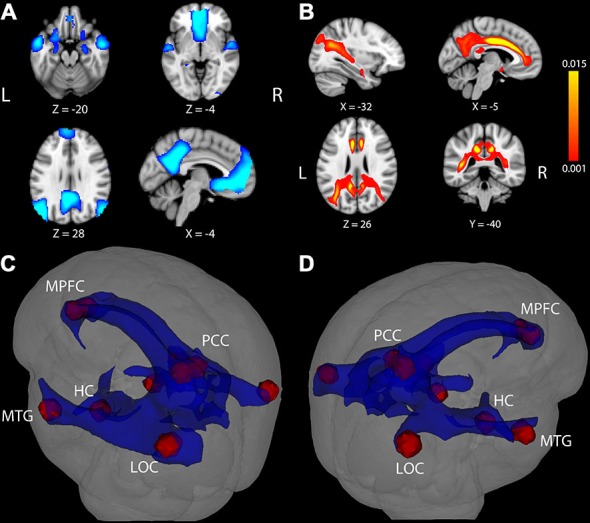
**Task-induced deactivation in the default mode network (DMN) and DMN white matter (WM) pathways. (A)** Regions showing significant deactivation at FWE-corrected *p* < 0.05. **(B)** Statistical map of probabilistic tractography indicating the proportion of streamlines passing through each voxel. The scale indicates a minimum value of 0.001 (0.1%) of all attempted streamlines passing through a given voxel, while the maximum was set at 0.015 (1.5%) of all attempted streamlines passing through a given voxel. Coordinate below each image is the MNI coordinate for that slice. **(C,D)** WM pathways (blue) connecting DMN regions (red) after averaging the entire group and thresholding at 0.1% of all streamlines attempted passing through a voxel. The WM pathways shown in blue were used to extract fractional anisotropy (FA) in each participant. LOC, lateral occipital cortex; MTG, middle temporal gyrus; HC, hippocampus; PCC, posterior cingulate cortex; MPFC, medial prefrontal cortex.

Results from the voxelwise contrast of the baseline fixation condition compared to the single task condition across participants revealed deactivation in the PCC, medial prefrontal cortex (MPFC), and in bilateral portions of lateral occipital cortex (LOC), HC, and middle temporal gyrus (MTG; Figure 1A). Coordinates of peak voxels within each cluster are listed in Table 2. A DMN mask was generated surrounding peak coordinates within each cluster (as described in Defining a Common DMN Network).

**Table 2:** **Peak fMRI coordinates from single condition < fixation contrast**.

Region	*Z*-max	*X*	*Y*	*Z*
**Left Hemisphere**
*Hippocampus (HC)*	6.00	−26	−14	−20
*Middle Temporal Gyrus (MTG)*	10.29	−58	−2	−16
*Lateral Occipital Cortex (LOC)*	12.06	−44	−76	34
**Right Hemisphere**
*Hippocampus (HC)*	6.31	26	−10	−20
*Middle Temporal Gyrus (MTG)*	9.87	56	−2	−16
*Lateral Occipital Cortex (LOC)*	9.57	52	−72	30
**Midline**
*Posterior Cingulate Cortex (PCC)*	10.81	−6	−42	40
*Medial Prefrontal Cortex (MPFC)*	10.81	−6	54	2

Z-max represents the peak Z-value within the cluster. X, Y, and Z are the MNI coordinates of the peak voxel within the cluster.

### White Matter Tractography, FA and Age

Results of tractography analysis revealed a set of DMN WM paths containing connections between the fMRI cortical ROIs. This set of DMN WM paths was observed in every participant and group results showed that the paths traveled through several major WM tracts, most prominently through bilateral portions of the cingulum, superior longitudinal fasciculus, inferior longitudinal fasciculus, and fornix (Figures 1B–D). A “network-level” DMN-WM mask was generated based on peak effects within these paths (as described in Probabilistic Tractography). As expected, regression analysis indicated a significant correlation between age and FA in the DMN-WM mask (β = −0.52, *t* = −6.49 *p* < 0.001).

### Effects of Age, Task Condition, and their Interaction, on Deactivation Magnitude within the DMN

**Figure 2:**
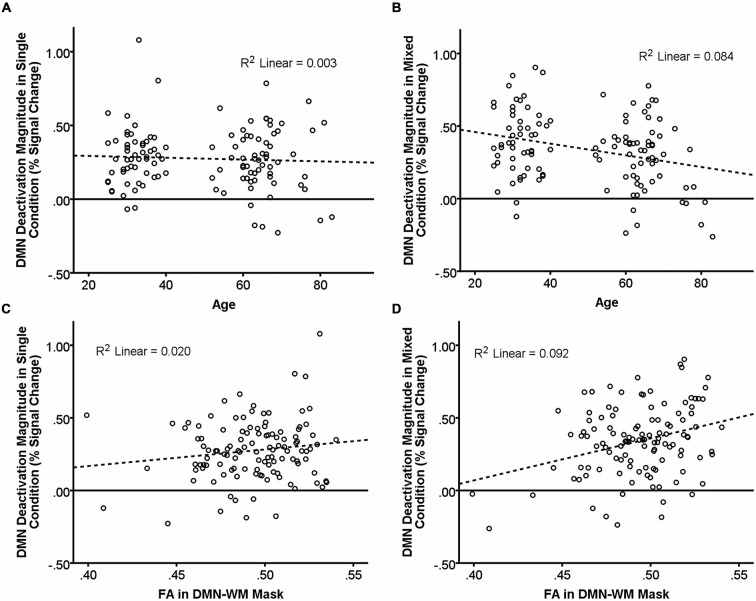
**Relationship of age and DMN WM microstructure with task-induced deactivation.** The scatter plots show DMN deactivation magnitude in the single and mixed conditions (greater positive values = greater deactivation) plotted against age **(A,B)** and FA in the DMN-WM mask **(C,D)**. Neither age **(A)** nor FA in the DMN-WM mask **(C)** correlated with task-induced deactivation in the single condition. Age **(B)** was negatively correlated and FA in the DMN-WM mask **(D)** was positively correlated with deactivation magnitude in the mixed condition. Dashed lines represent the linear best fit of the data. *R*^2^ values indicate the portion of the total variance explained by the line.

There were significant main effects of Age (β = −0.48, *t* = −2.56, *p* = 0.01) and Condition (β = 0.14, *t* = 3.47, *p* = 0.001) such that increasing age and the single condition were associated with less deactivation. There was also a significant Age × Condition interaction (β = −0.13, *t* = −3.16, *p* = 0.002). Examination of this interaction (Figures 2A,B) indicated that age was not related to DMN deactivation in the single condition (*p* = 0.58), but was significantly correlated in the mixed condition (β = −0.29, *t* = −3.24 *p* = 0.002).

### Effects of FA, Task Condition, and their Interaction, on Deactivation Magnitude within the DMN

Results indicated a main effect of Condition (β = 0.14, *t* = 3.41, *p* = 0.001) but not of FA (*p* = 0.320). There was, however, a significant FA × Condition interaction (β = 0.10, *t* = 2.33, *p* = 0.02). Examination of this interaction (Figures 2C,D) indicated that FA was not related to DMN deactivation in the single condition (*p* = 0.13), but was significantly correlated in the mixed condition (β = 0.30, *t* = 3.42 *p* = 0.001).

### Effects of Age, FA, and DMN Deactivation Magnitude on Task Performance

**Figure 3:**
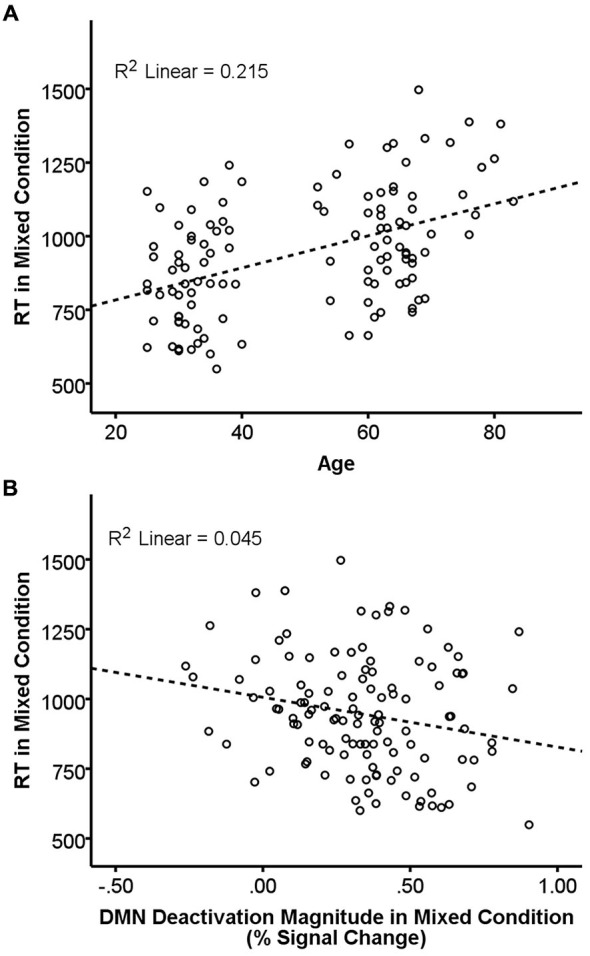
**Relationship of age and DMN deactivation magnitude with REACTION TIME (RT) in the mixed condition.** RT in the mixed condition was positively correlated with age **(A)** and negatively correlated with deactivation magnitude **(B)**. Dashed lines are the linear best-fit line. *R*^2^ is the portion of the total variance explained by the regression line.

Results of the GLMs examining the effects of age, FA, and deactivation magnitude on task performance are presented in Table 3. There were main effects of both age and FA in the DMN-WM mask on task performance (accuracy and RT). There was a significant Age × Condition interaction for accuracy. Examination of this interaction indicated that accuracy was only marginally correlated with age during the single condition (*p* = 0.09), but was significantly correlated with age during the mixed condition (β = −0.24, *t* = −2.68, *p* = 0.008). There were also marginal Age × Condition and Deactivation magnitude × Condition interactions for RT. Examination of these interactions (Figure 3) indicated that age was correlated with RT in both the single (β = 0.51, *t* = 6.38, *p* < 0.001) and mixed (β = 0.46, *t* = 5.62 *p* < 0.001) condition, while deactivation magnitude was correlated with RT in the mixed condition (β = −0.21, *t* = −2.32 *p* = 0.02) but not the single condition (*p* = 0.75).

**Table 3:** **GLM results for accuracy and reaction time**.

Accuracy			
Variable (*V*)	*V*	Condition	*V* × Condition
Age	β = −0.61, *t* = −3.17**	β = −0.30, *t* = −7.23***	β = −0.09, *t* = −2.19*
Deactivation	β = 0.09, *t* = 0.96	β = 0.32, *t* = 7.05***	β = 0.01, *t* = 0.21
Magnitude
FA in DMN-WM Mask	β = 0.43, *t* = 3.40***	β = −0.30, *t* = −7.10***	β = 0.03, *t* = 0.68

Reaction Time

**Variable** **(***V***)**	*V*	**Condition**	*V* × **Condition**

Age	β = 0.52, *t* = 4.90***	β = 0.53, *t* = 22.91***	β = 0.05, *t* = 1.97^∧^
Deactivation	β = 0.06, *t* = 1.22	β = 0.52, *t* = 21.41***	β = −0.05, *t* = −1.83^∧^
Magnitude
FA in DMN-WM Mask	β = −0.43, *t* = −6.72***	β = 0.53, *t* = 22.57***	β = −0.01, *t* = −0.53

V = Main effect of Variable, V × Condition = Interaction of variable and condition; ∧*p* < 0.10, *p < 0.05, **p < 0.005, ***p < 0.001.

### Mediation Analyses

Mediation analyses were performed to test the hypothesis that DMN WM microstructure accounts for the relationship between age and DMN deactivation magnitude in the mixed condition. Two mediation models were tested: Model (1) FA in the DMN-WM mask as the mediator of the relationship between age and DMN deactivation magnitude and Model (2) an alternative model with age as the mediator between the FA in the DMN-WM mask and DMN deactivation magnitude. The alternative model (Model 2) was chosen to test whether the FA in the DMN-WM mask and DMN deactivation were only related because both vary together with age, a condition which requires age to mediate the relationship between the other two variables (Salthouse, 2011).

**Figure 4:**
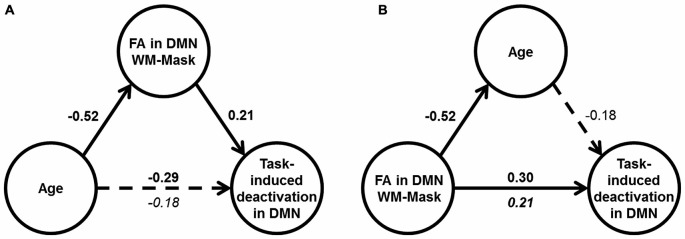
**Results of mediation analyses. (A)** Results of Model 1: Age correlated with DMN deactivation in the mixed condition (dotted arrow), but only the indirect effect through WM microstructure was significant (shown by solid arrows). **(B)** Results of Model 2: FA in DMN-WM mask had a direct effect on DMN deactivation in the mixed condition (solid arrow), and the indirect effect through age was non-significant (shown by dotted arrow). For **(A)** and **(B)**, values are standardized β-coefficients with significant β-values shown in bold. Total effect between the independent and dependent variable in the model are shown above the arrow, and the direct effect is shown beneath the arrow, *N* = 117.

Results of Model 1 (Figure 4A) indicated that the direct effect of age on magnitude of DMN deactivation (*c’* = −0.18, 95% CI [−0.38, 0.02]) was not significant, but instead was accounted for by the significant indirect pathway through FA in the DMN-WM mask (*ab* = −0.11, 95% CI [−0.24, −0.004]). Importantly, results of Model 2 (Figure 4B) indicated that the direct effect of FA in the DMN-WM mask on DMN deactivation (*c’* = 0.21, 95% CI [0.01, 0.41]) was not significantly mediated by age (*ab* = 0.09 [−0.002, 0.21]).

Due to the small gap between ages 40 and 50 in our sample, we repeated all analyses treating age as a dichotomous variable (<40 vs. >50). The results of these analyses revealed an identical pattern (same significant effects from GLMs and mediation analyses) as those described above with age as a continuous variable.

## Discussion

We found that individual differences in DMN deactivation magnitude are influenced by age and task condition, and that these relationships are mediated by WM microstructure. Extending previous findings, our fMRI results indicated interactive effects of age and condition such that increasing age affected deactivation magnitude during the more difficult (mixed) condition but not the easier (single) condition. In addition, we found that differences in WM microstructure accounted for a significant portion of the relationship between age and DMN deactivation magnitude in the more difficult condition. Our results suggest that age-related declines in WM microstructure contribute to functional regulation of DMN activity in older adults.

We observed functional deactivation in the PCC, MPFC, LOC, MTG, and HC across a participant sample ranging from 25 to 83 years of age. These regions represent a central portion of the DMN in studies of younger and older adult participant groups (Raichle et al., 2001; Grady et al., 2006; Persson et al., 2014). Overall task-induced deactivation magnitude across these regions was explored to investigate network-wide changes in DMN function associated with age and task performance. Results indicated relationships between task-induced deactivation magnitude, age and task performance. As expected, less deactivation was associated with both increasing age and poorer task performance. However, these main effects were qualified by an age by condition interaction, which indicated that increasing age was related to less DMN deactivation only in the mixed condition and had no effect on deactivation in the single condition.

In task-switching paradigms such as the present one, mixed conditions differ principally from single conditions in their increased emphasis on cognitive control processes such as switching and inhibitory control (Kramer et al., 1999; Kray and Lindenberger, 2000; Cepeda et al., 2001). The age by condition interaction in deactivation that we observed is thus consistent with a view that age-related declines in cognitive control are of high relevance to alterations in functional deactivation of DMN regions (Persson et al., 2007). The present findings are also consistent with results from previous studies using group comparisons between older and younger adults (Persson et al., 2007; Park et al., 2010; Sambataro et al., 2010). Importantly, all participants performed the mixed task with high accuracy (based on a median split, the older half of participants performed the mixed task with 94.6 ± 5.5% accuracy), which indicates that the lack of deactivation in the mixed condition is unlikely to be due to older adults not being actively engaged in the more difficult mixed task.

We next considered the potential association between DMN deactivation and WM microstructure. Probabilistic tractography was used to identify a set of WM pathways containing connections between cortical DMN structures commonly deactivated across our participant sample. As can be seen in Figure 1, the resulting WM network involved paths traveling through portions of midline tracts containing connections between medial temporal lobe and other limbic structures (bilateral portions of the cingulum and fornix) and long tracts containing connections between widely distributed neocortical structures (i.e., bilateral portions of the superior longitudinal fasciculus and inferior longitudinal fasciculus). Results from our tractography analysis are consistent with known anatomical connections between DMN cortical structures and show good correspondence with tracts identified in previous DMN structural connectivity studies conducted in separate groups of younger adults (Greicius et al., 2009; van den Heuvel et al., 2009; van Oort et al., 2014) and older adults (Teipel et al., 2010; Hahn et al., 2013; Weiler et al., 2014).

As expected, FA within the common network of DMN WM paths was negatively correlated with age. Interestingly, we found a significant FA by condition interaction, such that lower FA was associated with less deactivation magnitude during the mixed condition, but not associated with deactivation magnitude in the single condition. Several previous studies have reported correlations between FA and functional connectivity values within portions of the DMN (Andrews-Hanna et al., 2007; Teipel et al., 2010; van Oort et al., 2014). However, the present finding of an FA by condition interaction represents the first evidence to our knowledge indicating an increased association between WM microstructure and deactivation of the DMN as task demands increase.

Our findings thus suggest that age-related declines in WM microstructure may be associated with dynamic rather than static effects on functional DMN response during cognitive task processing. A different possible explanation for the present results could be that DMN structure and function may undergo independent changes in aging, which could reflect either distinct or shared dependence on some other unmeasured biological correlates of aging (Salthouse, 2011). Our use of continuous variables afforded the opportunity to test between these possibilities using mediation analyses. Results of mediation analyses cannot determine causality. Nevertheless, the overall pattern of results from our mediation analyses are more consistent with a view that DMN WM microstructure contributed to age-related functional dysregulation of DMN response observed in our study rather than some other (unmeasured) biological correlates of aging.

The finding that FA was related to DMN deactivation in the mixed condition but not the single condition has implications for behavioral results showing that older adults experience more interference from internally generated distraction than younger adults during cognitive control tasks (Healey et al., 2013). Our results suggest that age-related declines in WM microstructure within the DMN may contribute to such increased internal distraction in older adults during attention-demanding cognitive control processes. For instance, one possibility would be that intact WM connections between DMN regions may enable younger adults to efficiently regulate/reduce functional activity within the DMN in response to increased demands on cognitive control processes. In contrast, poorer WM microstructure within the DMN may decrease the capacity of older adults to dampen DMN functional activity when additional resources are required by the active task. The overall outcome would be relatively greater DMN functional activation and increased internally-generated distraction for older adults during attention-demanding active tasks.

Although our results indicated that WM microstructure attenuated a substantial proportion of the variance (38%) in the total relationship between age and task-induced deactivation, they also suggest that more than half of the variance is not accounted for by WM microstructure. There are likely to be many other neurobiological contributors to age-related declines in DMN function not tested here. Amyloid load may be especially relevant as it has been linked with altered DMN activity and functional connectivity (Celone et al., 2006; Hedden et al., 2009; Sperling et al., 2009; Sheline et al., 2010). Future work will be required to determine the separate and potentially synergistic contributions of WM microstructure and amyloid load to age-related changes in DMN functioning.

The present study has several caveats. First, in order to make stronger conclusions about the process of aging, it will be important to cross-validate these findings using a longitudinal design. Additionally, while DTI provides an indirect measure of WM microstructure, it does not directly measure axons or connections between regions. Rather, lower WM microstructure could contribute to age-related changes in functional deactivation in a number of ways. For example, alterations in WM connectivity would be expected to affect neurotransmitter signaling and recent evidence suggests that degree of DMN deactivation in younger adults is dependent on the balance between glutamate and GABA concentrations in the precuneus (Hu et al., 2013). While challenging, future research should attempt to integrate fMRI, DTI and spectroscopy and/or PET to identify potentially dissociable contributions of WM microstructure and neurotransmitter release on functional dysregulation of the DMN in aging. Finally, neither the roles of gray matter atrophy nor clinically silent pathologies were assessed in the present study. Previous studies have indicated that gray matter volume is not strongly correlated with deactivation magnitudes in older adults without clinical dementia (He et al., 2007; Damoiseaux et al., 2008; Threlkeld et al., 2011), and therefore likely has minimal effect. Silent pathologies such as microvascular insult and abnormal protein aggregation cannot be ruled out in the present sample, which could account for some of the age-related changes in deactivation. Future work should look to investigate the independent and synergistic roles of various clinically silent pathologies and WM microstructure in age-related functional changes in the DMN.

In conclusion, our results suggest that reductions in dynamic modulation of DMN activity are not simply an all-or-none phenomenon that begins in older age. Instead, our findings suggest that reduced functional modulation of DMN activity in response to increases in cognitive control demands appears to scale with increasing age. In addition, the present results suggest a contribution of WM microstructure to functional dysregulation of the DMN, thus providing evidence for one potential mechanism underlying the failure to modulate DMN deactivation in older adults.

## Author Contributions

CB and BG were involved in design, analysis, and interpretation of the work. JH, ZZ, and NF were involved in acquisition, analysis, and interpretation of the work. All authors were involved in drafting and revising the manuscript, have approved the final version, and agree to be accountable for all aspect of the accuracy and integrity of the work.

## Funding

This study was supported by a grant to BG from the National Institute on Aging of the National Institutes of Health under award number R01AG033036. CB received fellowship support from the National Center for Advancing Translational Sciences under award number TL1TR000115.

## Conflict of Interest Statement

The authors declare that the research was conducted in the absence of any commercial or financial relationships that could be construed as a potential conflict of interest.
